# Nanomaterials and the Precautionary Principle

**DOI:** 10.1289/ehp.1103687

**Published:** 2011-06

**Authors:** Kevin C. Elliott

**Affiliations:** Department of Philosophy and USC NanoCenter, University of South Carolina, Columbia, South Carolina, E-mail: ke@sc.edu

Kessler (2011) provided a valuable update on the current state of research and regulatory policy concerning nanomaterials. However, the article could give the misleading impression that the precautionary principle constitutes a straightforward guideline for improving public policy in this area. Instead, the precautionary principle provides only a general framework that must be specified before one can adequately assess its implications for policy.

Near the beginning of the article, Kessler (2011) quoted Alexis Baden-Mayer, who worried,

[I]n our regulation of food and consumer products, we don’t implement the precautionary principle. Things go to market before we know whether or not they’re really safe for human beings over the long term.

Kessler (2011) concluded with a quotation from Michael Hansen:

I think we need to take a precautionary approach because we’ve learned the hard way over and over and over again. You’d think we would learn.

By framing the issues in this way, Kessler (2011) intimated that the precautionary principle could serve as a valuable guide for future research and policy making. However, without further specification, the principle provides only a rough outlook or orientation rather than a specific regulatory plan of action; its merits cannot be clearly evaluated unless a number of further questions are answered.

A number of scholars have attempted to clarify how various formulations of the precautionary principle relate to one another. There are at least three important features that vary in different accounts of the principle: *a*) the threats that ought to be addressed; *b*) the amount and kinds of knowledge necessary to justify precautionary measures; and *c*) the specific precautionary measures that ought to be taken (Elliott 2010; Manson 2002; Sandin 1999). All three issues require further discussion in the case of nanomaterial research and regulation.

Regarding threats, one of the most crucial issues is whether it is sufficient to show that nanoparticles are safe for humans or whether they must also be shown to be safe for the environment—and, if so, what environmental impacts must be tested. Andrew Maynard hinted at this issue:

I think there is a greater chance that we’re going to see long-term environmental impacts from these materials than we are going to see short-term consumer impacts. (Kessler 2011)

Given the vast array of nanoparticles under consideration, it seems doubtful that they could all be thoroughly tested for a wide range of environmental effects before allowing their use.

This raises the question of how much evidence should be demanded before approving particular sorts of nanoparticles. A number of questions are relevant here, some of which are touched on by Kessler (2011): What kinds of screening studies should be required? When should *in vivo* studies be required? What structural or functional changes to a nanoparticle (e.g., size, crystal structure, manufacturing process) should trigger new toxicity studies? Should by-products of the production process also be studied in order to declare a nanoparticle safe (Templeton et al. 2006)? What steps must be taken to ensure that multiple manufacturing batches of the same nanoparticle result in products with the same toxicity profile? Does it matter what kinds of consumer products the nanoparticles are used for?

Finally, although many proponents and opponents of the precautionary principle treat the precautionary principle as if it requires bans on potential threats until they are shown to be safe, a range of other positions are also available on this issue. Three options include *a*) insisting that government agencies be notified when products contain particular nanoparticles; *b*) demanding labeling; or *c*) taking steps to minimize human or environmental exposure to nanoparticles until they have received further testing. Kessler (2011) highlighted our present failure to achieve some of these minimal steps.

These considerations do not by themselves count as sufficient reasons for rejecting the precautionary principle, but they do show that the decision to adopt it is the start of a complicated conversation rather than a straightforward choice about how to regulate nanomaterials.
